# Characterization of Integrons and Quinolone Resistance in Clinical *Escherichia coli* Isolates in Mansoura City, Egypt

**DOI:** 10.1155/2021/6468942

**Published:** 2021-09-04

**Authors:** Shaymaa H. Abdel-Rhman, Rehab M. Elbargisy, Dina E. Rizk

**Affiliations:** ^1^Microbiology and Immunology Department, Faculty of Pharmacy, Mansoura University, Mansoura, Egypt; ^2^Department of Pharmaceutics and Pharmaceutical Biotechnology, Faculty of Pharmacy, Taibah University, AlMadinah Al Munawwarah, Saudi Arabia; ^3^Department of Pharmaceutics, College of Pharmacy, Jouf University, Sakaka, Al-Jouf, Saudi Arabia

## Abstract

*Escherichia coli* is a common pathogen in both humans and animals. Quinolones are used to treat infections caused by Gram-negative bacteria, but resistance genes emerged. Only scarce studies investigated the association between plasmid-mediated quinolone resistance (PMQR) genes and integrons in clinical isolates of *E. coli*. The current study investigated the prevalence of quinolone resistance and integrons among 134 clinical *E. coli* isolates. Eighty (59.70%) isolates were quinolone-resistant, and 60/134 (44.77%) isolates were integron positive with the predominance of class I integrons (98.33%). There was a significant association between quinolone resistance and the presence of integrons (*P* < 0.0001). Isolates from Urology and Nephrology Center and Gastroenterology Hospital were significantly quinolone-resistant and integron positive (*P* ≤ 0.0005). Detection of PMQR genes on plasmids of integron-positive isolates showed that the active efflux pump genes *oqxAB* and *qepA* had the highest prevalence (72.22%), followed by the aminoglycoside acetyltransferase gene (*aac(6′)-Ib-cr*, 66.67%) and the quinolone resistance genes (*qnr*, 61.11%). Amplification and sequencing of integrons' variable regions illustrated that no quinolone resistance genes were detected, and the most predominant gene cassettes were for trimethoprim and aminoglycoside resistance including *dfrA17, dfrB4*, and *dfrA17-aadA5*. In conclusion, this study reported the high prevalence of PMQR genes and integrons among clinical *E. coli* isolates. Although PMQR genes are not cassette-born, they were associated with integrons' presence, which contributes to the widespread of quinolone resistance in Egypt.

## 1. Introduction

Despite being a well-known member of the normal flora for both humans and animals [1], the pathogenic strains of *Escherichia coli* cause a wide variety of infections including the skin and soft tissue, urinary tract, gastrointestinal tract, and central nervous system [2]. The severity of infections can vary from mild to life-threatening conditions based on the virulence capacity and antimicrobial resistance of the bacterium.

Fluoroquinolones are broad-spectrum synthetic antibiotics that can successfully treat infections caused by *Enterobacteriaceae*. Unfortunately, resistance to fluoroquinolones is increasing that is mediated chromosomally by modifications in their targets such as DNA gyrase and topoisomerase IV [3]. Plasmid-mediated quinolone resistance (PMQR) is now spreading among Gram-negative bacteria. Three main mechanisms are employed for PMQR resistance. The first is the modification of quinolones' targets by qnr proteins [encoded by quinolone resistance genes (*qnr* genes)]. Secondly, fluoroquinolones are modified through acetylation by the aminoglycoside acetyltransferase enzyme (encoded by the *aac(6*′*)-Ib-cr* gene). Lastly, excretion of hydrophobic fluoroquinolones by active efflux pumps such as QepA and OqxAB (encoded by *qep, oqxA*, and *oqxB* genes) [4].

Integrons are genetic elements that account for the dissemination of antimicrobial resistance genes in different species of bacteria. They are found on plasmids or transposons, which facilitate their transfer between bacterial cells [5]. Two main parts constitute an integron. The first part is made up of an integrase gene (*intI*), an integration site (attI), and a promoter. The second part includes an attachment site (attC) for a gene cassette. This cassette has a single gene and mostly an open reading frame but no promoter. When attI and attC sites recombine, genes carried by gene cassettes can be expressed through the integron promoter [6]. Gene cassettes integrated into integrons carry antimicrobial resistance genes that facilitate dissemination of antimicrobial resistance upon transfer of plasmids or transposons that include the integrons [7, 8]. Although different classes of integrons have been discussed before, class I and II integrons remain the most prevalent ones among resistant clinical isolates [9, 10].

Although the association between PMQR and integrons among environmental isolates has been reported in several studies [8, 11–13], only few studies investigated the association between them in clinical *E. coli* isolates, and the investigations of the location of these genes on the variable region gene cassettes were scarce. Therefore, our study aimed to investigate the prevalence and association of quinolone resistance and integrons among clinical *E. coli* isolates. Additionally, the prevalence of PMQR genes among integron-positive isolates was studied.

## 2. Materials and Methods

### 2.1. Bacterial Isolates

During the period from February to August 2019, a total of 134 nonreplicate clinical *E. coli* isolates were obtained from different clinical specimens [urine (72 isolates), throat swabs (2 isolates), stool (51 isolates), blood (1 isolate), and wound exudate (8 isolates)]. They were collected from 5 different hospitals located in Mansoura city, Egypt [Urology and Nephrology Center (UNC), Gastroenterology Hospital (GEH), Microbiology Diagnostic Infection Control Unit (MDICU), Specialized Medical Hospital (SMC), and Mansoura University Children Hospital (MUCH)]. Identification of bacterial isolates was done according to laboratory biochemical standard methods [14]. The study fulfills the ethical guidelines approved by “The Research Ethics Committee, Faculty of Pharmacy, Mansoura University” that follow the Code of Ethics of World Medical Association (Declaration of Helsinki involving the use and handling of human subjects; Code Number: 2021-9).

### 2.2. Determination of Quinolone Resistance

Resistance of the isolates to four quinolone antibiotics including ciprofloxacin (10 *μ*g), levofloxacin (5 *μ*g), norfloxacin (10 *μ*g), and ofloxacin (5 *μ*g) was determined using the standard Kirby–Bauer disk diffusion method following the CLSI guidelines [15].

### 2.3. Genomic and Plasmid DNA Extraction

Genomic DNA was obtained from all isolates using the modified boiling technique [16]. The filtrate was stored at −20°C and used as a template for polymerase chain reaction (PCR) amplification of integrase genes.

The selection of isolates for plasmid DNA extraction was based on the results obtained from PCR amplification of integrons on total DNA in addition to quinolone sensitivity testing. Plasmid DNA extraction was done using the GeneJET Plasmid Miniprep kit (K0502, EU, Lithuania).

### 2.4. PCR Amplification of Integrase Genes, Gene Cassettes, and (PMQR) Genes

Multiplex PCR was used for the detection of class I, II, and III integrons in all isolates using primers intI1, intI2, and intI3, respectively (Table 1). The variable regions of class I and II integrons and PMQR genes were amplified by PCR on plasmids extracted from selected 43 isolates using the primers listed in Table 1. PCR reaction mixture and cycling conditions were performed as previously described.

### 2.5. Restriction Fragment Length Polymorphism (RFLP) and Characterization of Gene Cassettes

To investigate the similarity between the gene cassettes of the different isolates, RFLP of purified PCR products of variable regions of class I and II integrons (extracted from the agarose gel using GenJet Gel extraction kit (K0691, EU, Lithuania)) was detected after digestion by restriction enzyme *HinfI* (CutSmart, New England Biolabs) [21] and sequenced on an automated sequencer (ABI Prism 3100). The resulted sequences were assembled using the CodonCode Aligner (version 9.0.1). BLAST (http://blast.ncbi.nlm.nih.gov/Blast.cgi) against the GenBank database was performed for sequence comparison and annotation.

### 2.6. Nucleotide Sequence Accession Numbers

The integron gene cassette nucleotide sequences identified in this study have been recorded in the GenBank database (GenBank accessions no. MW770320 to MW770348).

### 2.7. Statistical Analysis

GraphPad InStat (Fisher's exact and Chi-square tests) was used to analyze the results statistically. Results with *P*-value ≤0.05 were considered of statistical significance.

## 3. Results

### 3.1. Resistance to Quinolones

The results of the antimicrobial sensitivity test illustrated that resistance to tested quinolones was significantly prevalent among isolates. It was found that 80 (59.70%), 79 (58.95%), 78 (58.20%), and 77 (57.46%) of isolates were resistant to norfloxacin, ciprofloxacin, ofloxacin, and levofloxacin, respectively.

It was found that urine isolates were significantly resistant to the tested quinolones (*P* < 0.0001). Besides, these isolates were significantly associated with UNC (*P* < 0.0001) and GEH (*P*=0.0005).

### 3.2. PCR Amplification of Integrase Genes, Gene Cassettes, and PMQR Genes

Detection of integrase genes on genomic DNA showed that 60 isolates (44.8%) harbored the genes. Integron I was significantly prevalent (59 isolates, 98.33%; *P* < 0.0001), followed by integron II (5 isolates (8.33%), 4 of them coexist with integrons I) and integron III [3 isolates (5%) that have also integron I].

Generally, integrons were significantly detected in isolates from UNC and GEH (*P* < 0.0001). A significant association of quinolone resistance and integrons was observed (*P* value = 0.0061), where 43/80 (53.75%) of quinolone-resistant isolates harbored integrons genes. These 43 isolates were significantly associated with UNC and GEH (*P* < 0.0001). All urine isolates having integrons were also quinolone-resistant (*P* < 0.0001).

PMQR genes were detected on plasmid DNA of the 36/43 isolates (83.72%) that showed phenotypic quinolone resistance and harbored integrons. The results in Table 2 showed that *aac*(*6′*)-*Ib*, *oqxB*, *qnrS*, and *qepA* were the most prevalent genes. None of the test isolates carried the *qnrC* gene. Seven isolates did not harbor any of the tested genes. The distribution of PMQR genes among different sources was illustrated in Table 2. It showed that all genes were significantly detected in urine samples (*P* < 0.0001), while isolates from a throat swab and blood carried one and two genes, respectively.

**Figure 1:**
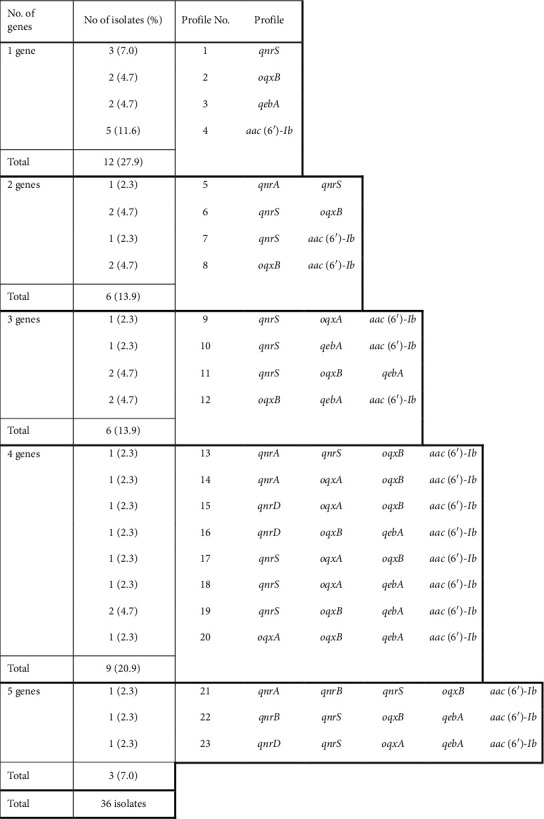
The gene profile of PMQR genes among the 36 clinical *E. coli* isolates.

Figure 1 illustrates the obtained 23 gene profiles. Isolates that carried 5 genes were all isolated from urine samples from UNC. Nine profiles were shown by more than 1 isolate (Pr1–Pr4, Pr6, Pr8, Pr11, Pr12, and Pr19). The most predominant profile was Pr4 (5 isolates, 11.62%). *qnrA*, *qnrB*, *qnrD*, and *oqxA* genes were not detected as a single-gene profile. The *qnrB* gene was associated with profiles that carried 5 genes. The genes coexisted with each other without any significant association.

### 3.3. Integrons and Characterization of Gene Cassettes

The sizes of the integron gene cassettes and their distribution among tested isolates are shown in Table 3. The investigation of integron gene cassettes revealed that integron I variable regions were amplified in 31/42 isolates (73.81%), where 27 isolates showed 1 variable region with sizes ranging from 350 bp to 2,200 bp. Integron II variable regions with a size of 2,200 bp were amplified in 2/4 (50%) isolates. Variable regions of 1,600 bp (*P* < 0.0001) and 600 bp were more prevalent among isolates.

**Figure 2:**
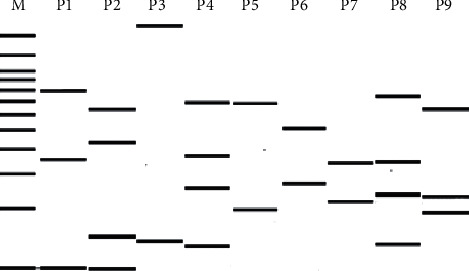
Schematic representation of the patterns obtained by digestion of integron I and II regions by *HinfI* restriction enzyme. M: 100 bp DNA marker, P1: 2,200 bp, P2 and P3: 1,800 bp, P4: 1,600 bp, P5: 1,000 bp, P6: 750 bp and 850 bp, P7: 600 bp, and P8 and P9: 2,200 bp.

The digestion of the amplified variable regions of integrons I and II with *HinfI* restriction enzyme resulted in seven different patterns (P1–P7) and two different patterns (P8 and P9), respectively.  Figure 2 illustrates the restriction patterns corresponding to each size. The variable region of 1,600 bp gave the pattern P4 and was found in eighteen isolates. P7 that represents a 600 bp amplicon was found in eight isolates.

Gene sequencing of the amplified variable regions showed that seven different gene cassettes were detected among 23 class I integron-positive isolates and two gene cassettes in 2/4 of class II integron-positive isolates. Five gene cassettes had one gene either of dihydrofolate reductase (*dfr* family) or aminoglycosides resistance (*aad* family). Three gene cassettes included both *dfr* and *aad* genes.

The detected genes belonged to trimethoprim resistance (*dfrA1*, *dfrA7*, *dfrA12*, *dfrA17*, and *dfrB4*), aminoglycosides resistance (*aadA1*, *aadA2*, *aadA5*, and *aadA22*), and streptothricin resistance (*sat2*; streptothricin acetyltransferase). There was also an open reading frame of unknown function (*orfF*).

The most common gene cassette was *dfrA17* (*n* = 9, 31.03%), followed by *dfrB4* (*n* = 8, 27.58%), *dfrA17-aadA5* (*n* = 5, 17.24%), *aadA2-orfF-dfrA12* (*n* = 2, 6.89%), and *aadA22* (*n* = 2, 6.89%). *dfrA1-sat2-aadA1*, *dfrA7*, and *drfA12* gene cassettes were represented by one isolate each (3.44%). *dfrA17-aadA5* gene cassette was detected in integrons I and II (Table 4).

Table 5 shows the characterization of the 43 *E. coli* isolates including the isolation source, gene profiles, type of integron, and gene cassettes. It illustrated that the isolate may have more than one variable region with different gene cassettes. Moreover, it showed that the variable regions of approximately 1,600 bp were similar as they carry either (*dfrA17-aadA5*) or *dfrA17*. Integron-I-positive isolates that carried the *dfrA17* gene cassette also harbored the *aadA5* gene cassette that had a frameshift mutation by addition and deletion of several nucleotides, which resulted in several internal stop codons and gaps.

## 4. Discussion

Since the discovery of PMQR genes in 1998, a wide distribution of these determinants, especially in members of *Enterobacteriaceae*, was observed. This may be due to the broad use of fluoroquinolone antibiotics in human medicine, veterinary medicine, and the environment [22]. In the current study, fluoroquinolones' resistance was highly prevalent in the tested isolates (57.46–59.70%). A similar result was reported by a study performed in Egypt [23]. In contrast, previous studies from several countries [24–28] reported that most of the tested isolates were susceptible to fluoroquinolones (70–90%). The main causes behind antibiotic resistance are the misuse of antibiotics, improper prescription by physicians, and unawareness of patients who do not follow dosage regimens [29]. Furthermore, the use of quinolones in agriculture may account for an increase in PMQR genes in *Enterobacteriaceae* [30]. Fluoroquinolone resistance and *E. coli* isolated from urine were found to have a significant correlation, where fluoroquinolones were one of the most important therapeutic regimens used in the treatment of uropathogens [31]. Therefore, this may explain the high prevalence of fluoroquinolone resistance among isolates from urinary tract infections, particularly in developing countries.

Integrons have been described as an acquired resistance mechanism by capturing, excising, and expressing gene cassettes through site-specific recombination, thus aiding in the spread of antibiotic resistance [32]. In this study, integrons were found in 44.77% of the tested isolates, which is similar to other studies [10, 12]. Several studies, on the other hand, found a higher prevalence rate of integrons [33–35]. The present study revealed the predominance of integron I among quinolone-resistant isolates (59/80 isolates, 73.75%). In previous studies, integron I was more frequent among Gram-negative bacteria [36–38].

PMQR was detected in 36/43 (83.72%) phenotypically fluoroquinolone-resistant isolates. Several studies showed similar results [39, 40]. Other studies that investigated PMQR genes recorded variations in their prevalence rate even in the same country [41–44]. This variation may be due to the difference in study populations, types of isolates, selection criteria, and geographical distribution [45].

The active efflux pump genes, *oqxAB* and *qepA*, had the highest prevalence (72.22%), followed by *aac(6′)-Ib-cr* gene (66.67%) and *qnr* genes (61.11%). A study conducted in Egypt recorded *aac(6′)-Ib-cr* as the most frequent PMQR gene (61%), followed by *qnrS* (43.3%) [39]. Another study in China indicated a similar incidence rate of *acc(6′)-Ib-cr* and *qepA* genes [46]. Among the detected *qnr* genes, *qnrS* showed the highest prevalence, which agrees with previous studies [47–49]. Although detected in only 4.65% of our isolates, *qnrB* gene was reported as one of the most abundant genes in other studies [39, 45, 50]. *qnrC* gene was absent in all tested isolates, which agrees with other studies [45, 51, 52]. The *qepA* distribution rate in our results was similar to what was reported [52]. Several studies reported that *aac(6′)-Ib-cr* was detected more than *qnr* genes in *Enterobacteriac*eae [39, 44, 53–55]. This may be attributed to the gene coding for this enzyme is highly prevalent in fluoroquinolone-resistant phenotypes. Besides, upon exposure to ciprofloxacin, this enzyme eases the selection of high-resistant ciprofloxacin chromosomal mutants. In addition, the low level of fluoroquinolone resistance mediated by this enzyme can be converted to high-level resistance when coexisting with qnr proteins in the same bacterial isolates [56]. Seven isolates that showed quinolone resistance phenotypically did not harbor any of the tested genes on their plasmid. Their quinolone resistance may be attributed to other mechanisms that were not investigated in this study like chromosomal mutations of *gyrA* or *parC* genes or due to impermeability of the membranes [39].

In the current study, the 36 isolates harboring the PMQR genes showed 23 different gene profiles. This result was consistent with what was reported earlier [49, 57]. Concerning the coexistence of PMQR genes, no significant association was found. Similar results were reported previously [49, 58]. Most isolates (23/36 isolates, 63.88%) showed more than 1 mechanism of PMQR, where 36.11% (13/36 isolates) of them had genes that encode the 3 PMQR mechanisms. A recent study in China has demonstrated the coexistence of three PMQR genes, including *aac(6′)-Ib-cr*, *qnrS2*, and *oqxAB*, on a multiple resistance plasmid [59]. A significant association was found between resistance mechanisms encoding efflux with *qnr* and *aac(6′)-Ib-cr* genes (*P*-value = 0.0179 and 0.0023, respectively). No significant association between resistance mechanisms encoded by *aac(6′)-Ib-cr* and *qnr* genes was found, which contrasts with other studies [39, 60].

Among the 43 integron-positive *E. coli* isolates, gene cassettes were amplified in 32 isolates (74.41%), which is comparable to the results reported by Zhang et al. (72.7%) [61]. Although the isolates carried the integrase genes, the gene cassettes were not amplified in 26.19% and 50% of class I and II integron positive isolates, respectively. The lack or variation of a 3′-conserved segment [62] and the variation in the binding site of the primer or the extensive size of gene cassette [63, 64] may explain the absence of gene cassettes in these isolates. The most abundant variable regions were of size 600 and 1,600 bp as they were found in 8 and 18 isolates, respectively. Similar results were reported earlier [65,66]. Identical restriction patterns of the same sized variable regions may be indicative of the presence of the same gene cassettes that were confirmed by sequencing.

Several gene cassettes belonging to class I integron have been described. Resistance to several antibiotics is mainly due to these cassettes [67]. In the current study, 23 out of 42 class I integron positive isolates carried 7 different gene cassettes (*dfrA7*, *dfrA12*, *dfrA17*, *dfrB4*, *aadA22*, *dfrA17-aadA5*, and *aadA2-orfF-dfrA12*). The reported cassettes encode for resistance to trimethoprim and aminoglycosides and have been recorded among class I integrons in previous studies [10, 13, 61, 68, 69]. The diversity of class I integron cassettes is comparable to previous studies [10, 61] that reported 10 and 7 different gene cassettes among the tested isolates, respectively. In contrast to class I integrons, few different resistance cassettes have been associated with class II integrons [70]. In class II integron positive isolates of the current study, *dfrA17-aadA5* and *dfrA1-sat2-aadA1* gene cassettes were found. Zhang et al. [61] reported *dfrA1-sat2-aadA1* as a single gene cassette among class II integron positive isolates.

The gene cassettes in the present study showed the dominance of *dfr* genes within class I and II integrons. Similar results were reported previously [10, 34, 69]. This may be explained by the extensive use of trimethoprim as a first-line treatment for urinary tract infections in both hospital and community settings [34]. *dfrA17*, *dfrB4*, and *dfrA17-aadA5* gene cassettes were found in nine, eight, and five isolates, respectively. This may be due to the interspecies transfer of the integrons. Integrons disseminate in hospitals through the cross-transmission of integron-carrying clones from one patient to another [34, 71].

Concerning the variable region of 1,600 bp, four and seven variable regions carried *dfrA17-aadA5* and *dfrA17* gene cassettes, respectively. It is worth mentioning that these seven variable regions had also the *aadA5* gene, which had a frameshift mutation that resulted in several internal stop codons and gaps. Similar results were reported by Chen et al. [66] where the 1,600 bp amplicon harbored *dfrA17*-*aadA5* genes. This cassette array was reported in other bacteria and other regions around the world [9, 72–75]. This array can spread all over the world by self-transferable plasmids in humans or animals [76].

No PMQR genes were detected in the amplified gene cassettes. This indicates that quinolone resistance genes are not cassette-born. This coincides with the study of Kubomura et al. who found that except for *dfrA* and *aadA* genes, the antibiotic resistance genes are mostly found outside the integrons [69]. This may also suggest that resistance is related to other mobile genetic elements such as plasmids and transposons [77].

## 5. Conclusion

This study indicates the high prevalence of quinolone resistance and integrons in *E. coli* isolates, especially those from urine. PMQR genes were widely distributed among the tested isolates, implying lateral gene transfer. Gene sequencing of the amplified variable regions of integrons revealed that the most predominant gene cassettes were for trimethoprim and aminoglycoside resistance. Although PMQR genes are not cassette-born, they were associated with integrons' presence on the plasmids. Future studies to explain this association phenomenon should be performed. Continued surveillance of PMQR and integrons should be conducted to control their spread and the associated health risks.

## Figures and Tables

**Table 1:** Primer sequences and conditions used in PCR amplification.

Target gene	Sequence 5′–3′		Annealing temperature (°C)	Product size (bp)	Reference
*qnrA*	F	AGAGGATTTCTCACGCCAGG	60	580	[17]
	R	TGCCAGGCACAGATCTTGAC			

*qnrB*	F	GGMATHGAAATTCGCCACTG	60	264	[17]
	R	TTTGCYGYYCGCCAGTCGAA			

*qnrC*	F	GGGTTGTACATTTATTGAATC	52	307	[18]
	R	TCCACTTTACGAGGTTCT			

*qnrD*	F	CGAGATCAATTTACGGGGAATA	52	465	[19]
	R	AACAAGCTGAAGCGCCTG			

*qnrS*	F	GCAAGTTCATTGAACAGGCT	60	428	[17]
	R	TCTAAACCGTCGAGTTCGGCG			

*qepA*	F	CTGCAGGTACTGCGTCATG	52	403	[20]
	R	CGTGTTGCTGGAGTTCTTC			

*oqxA*	F	GACAGCGTCGCACAGAATG	57	339	[20]
	R	GGAGACGAGGTTGGTATGGA			

*oqxB*	F	CGAAGAAAGACCTCCCTACCC	57	240	[20]
	R	CGCCGCCAATGAGATACA			

*aac(6*′*)-Ib*	F	TTGCGATGCTCTATGAGTGGCTA	60	482	[18]
	R	CTCGAATGCCTGGCGTGTTT			

*IntI1*	F	GGTCAAGGATCTGGATTTCG	60	436	[21]
	R	ACATGCGTGTAAATCATCGTC			

*IntI2*	F	CACGGATATGCGACAAAAAGG	60	788	[21]
	R	TGTAGCAAACGAGTGACGAAATG			

*IntI3*	F	AGTGGGTGGCGAATGAGTG	60	600	[21]
	R	TGTTCTTGTATCGGCAGGTG			

*5*′*CS*	F	GGCATCCAAGCAGCAAG	58	Variable	[21]
*3*′*CS*	R	AAGCAGACTTGACCTGA			

*attI2-F*	F	GACGGCATGCACGATTTGTA	58	Variable	[21]
*orfX-R*	R	GATGCCATCGCAAGTACGAG			

F: forward and R: reverse.

**Table 2:** The PMQR genes distribution among different sources.

Source (no. of isolates)	*qnrA*	*qnrB*	*qnrC*	*qnrD*	*qnrS*	*oqxA*	*oqxB*	*qepA*	*aac(6*′*)-Ib*
Urine (32)	3	2	—	3	12^*∗∗*^	6	15^*∗∗∗*^	12^*∗∗∗*^	19^*∗∗∗*^
Stool (5)	—	—	—	—	3	1	3	2	4
Wound (3)	—	—	—	—	2	—	2	—	1
Throat swab (2)	—	—	—	—	1	—	—	—	—
Blood (1)	1	—	—	—	1	—	—	—	—
Total (43)	4 (9.30%)	2 (4.65%)	0 (0%)	3 (6.97%)	19 (44.19%)	7 (16.28%)	20 (46.51%)	14 (32.56%)	24 (55.81%)

^*∗∗*^*P*-value = 0.004 and ^*∗∗∗*^*P* < 0.0001.

**Table 3:** The distribution of integron variable region's sizes among tested *E. coli* isolates.

Band no. (B)		Variable regions' size (bp)	No. of isolates (%)
Class I integron	1	2,200	1 (2.38)
	2	1,800	2 (4.76)
	3	1,600	**15 (35.71)**
	4	1,000	1 (2.38)
	5	850	1 (2.38)
	6	750	1 (2.38)
	7	600	**5 (11.90)**
	8	350	1 (2.38)
	9	1,000, 750	1 (2.38)
	10	1,600, 600	2 (4.76)
	11	1,600, 850, 600	1 (2.38)
	12	Without PCR products	11 (26.19)
	Total		42 (100)

Class II integron	1	2,200	2 (50)
	2	Without PCR products	2 (50)
	Total		4 (100)

**Table 4:** Size and number of gene cassettes amplified from integron-positive *E. coli* isolates' variable region.

Gene cassette (*s*)	Size (bp)	No. of isolates (%)
Class I integron		
*dfrB4*	566	4 (13.79)
	565	1 (3.44)
	563	2 (6.89)
	853	1 (3.44)
*dfrA7*	775	1 (3.44)
*dfrA12*	1,828	1 (3.44)
*dfrA17*	362	1 (3.44)
	785	1 (3.44)
	1,615	1 (3.44)
	1,618	1 (3.44)
	1,619	1 (3.44)
	1,624	1 (3.44)
	1,625	1 (3.44)
	1,642	1 (3.44)
	1,675	1 (3.44)
*aadA22*	1,027	1 (3.44)
	1,032	1 (3.44)
*dfrA17-aadA5*	1,622	1 (3.44)
	1,628	1 (3.44)
	1,632	1 (3.44)
	2,292	1 (3.44)
*aadA2-hypothetical protein (orfF)-dfrA12*	1,863	1 (3.44)
	2,142	1 (3.44)

Class II integron		
*dfrA17-aadA5*	2,169	1 (3.44)
*dfrA1-sat2-aadA1*	2,172	1 (3.44)
Total		29

**Table 5:** Characterization of 43 quinolone-resistant integron-positive clinical *E. coli* isolates.

Isolate	Ward	Source	PMQR genes	No. of genes	Integrons	Variable regions size (bp)	Gene cassette (s)	No. of gene cassettes	Accession number
1	UNC	Urine	*aac(6*′*)-Ib*	3	I	—	—	—	—

2	GEH	Wound	*qnrS*	1	I	—	—	—	—

3	MDICU	Stool	*qnrS*, *oqxA*, *oqxB*, and *aac(6*′*)-Ib*	4	I II	785 —	*dfrA17* —	1	MW770328

4	GEH	Wound	*oqxB* and *aac(6*′*)-Ib*	2	I	1,600	‐^1^	—	—

10	UNC	Urine	—	—	I	—	—	—	—

14	GEH	Wound	*qnrS* and *oqxB*	2	I	2,144	*aadA2-(orfF)-dfrA12*	3	MW770345

15	UNC	Urine	*qnrA*, *qnrS*, *oqxB*, and *aac(6*′*)-Ib*	4	I	1,632	*dfrA17-aadA5*	2	MW770333

17	UNC	Urine	*qnrD*, *oqxB*, *qepA*, and *aac(6*′*)-Ib*	4	I	566	*dfrB4*	1	MW770321

20	GEH	Throat swab	—	—	I	—	—	—	—

23	GEH	Blood	*qnrA* and *qnrS*	2	I	1,600	‐^1^	—	—

24	GEH	Throat swab	*qnrS*	1	I	1,826	*aadA2-(orfF)-dfrA12*	3	MW770343

26	MDICU	Urine	*qnrD*, *oqxA*, *oqxB*, and *aac(6*′*)-Ib*	4	I	1,628	*aadA5-dfrA17*	2	MW770334

30	UNC	Urine	*qnrS* and *oqxB*	2	I	775 1,027	*dfrA7* *aadA22*	1 1	MW770329 MW770331

31	UNC	Urine	*qnrA*, *qnrB*, *qnrS*, *oqxB*, and *aac(6*′*)-Ib*	5	I	1,615	*dfrA17*	1	MW770335

32	UNC	Urine	*oqxB*, *qepA*, and *aac(6*′*)-Ib*	3	I III	2,292 —	*dfrA17-aadA5* —	2	MW770346

33	UNC	Urine	*qnrB*, *qnrS*, *oqxB*, and *qepA, aac(6*′*)-Ib*	5	I III	1,600 —	*-*^1^ —	—	—

38	UNC	Urine	*qnrB*, *qnrS*, *oqxB*, *qepA*, and *aac(6*′*)-Ib*	5	I	1,675	*dfrA17*	1	MW770342

40	MDICU	Urine	*qnrS*, *oqxB*, and *qepA*	3	I	1,600	*-* ^1^	—	—

44	UNC	Urine	—	—	I	—	—	—	—

45	UNC	Urine	*qnrS* and *aac(6*′*)-Ib*	2	I	566 1,622	*dfrB4* *aadA5-dfrA17*	1	MW770322 MW770336

48	MDICU	Urine	*aac(6*′*)-Ib*	1	I	1,600	*-* ^1^	—	—

51	UNC	Urine	*qnrD*, *qnrS*, *oqxA*, *qepA*, and *aac(6*′*)-Ib*	5	I II	1,618 2,172	*dfrA17* *dfrA1-sat2-aadA1*	1 3	MW770337 MW770347

53	UNC	Urine	*qnrS*, *oqxA*, *qepA*, and *aac(6*′*)-Ib*	4	I II	1,619 —	*dfrA17* —	1	MW770338

54	UNC	Urine	*aac(6*′*)-Ib*	1	I II	— 2,169	— *dfrA17-aadA5*	2	MW770348

56	UNC	Urine	*aac(6*′*)-Ib*	1	II	—	—	—	—

57	UNC	Urine	—	—	I	—	—	—	—

63	UNC	Urine	*aac(6*′*)-Ib*	1	I	1,828	*dfrA12*	1	MW770344

64	UNC	Urine	*qepA*	1	I	—	—	—	—

66	UNC	Urine	*oqxB*, *qepA*, and *aac(6*′*)-Ib*	3	I	1,032	*aadA22*	1	MW770332

73	GEH	Stool	*oqxB*	1	I	1,600	*-* ^1^	—	—

74	GEH	Stool	*qnrS*, *oqxB*, *qepA*, and *aac(6*′*)-Ib*	4	I	566 1,624	*dfrB4* *dfrA17*	1 1	MW770323 MW770339

84	UNC	Urine	*oqxA*, *oqxB*, *qepA*, and *aac(6*′*)-Ib*	4	I	—	—	—	—

85	GEH	Stool	*aac(6*′*)-Ib*	1	I	600	*-* ^1^	—	—

87	UNC	Urine	—	—	I	563 856 1,642	*dfrB4* *dfrB4* *dfrA17*	1 1 1	MW770324 MW770330 MW770340

89	UNC	Urine	*qnrS*, *oqxB*, and *qepA*	3	I	362	*dfrA17*	1	MW770320

94	UNC	Urine	—	—	I	—	—	—	—

95	UNC	Urine	—	—	I	1,600	*-* ^1^	—	—

106	UNC	Urine	*qnrA*, *oqxA*, *oqxB*, and *aac(6*′*)-Ib*	4	I	1,625	*dfrA17*	1	MW770341

107	UNC	Urine	*oqxB*	1	I	563	*dfrB4*	1	MW770325

110	UNC	Urine	*oqxB* and *aac(6*′*)-Ib*	2	I	—	—	—	—

111	UNC	Urine	*qepA*	1	I	1,600	*-* ^1^	—	—

116	GEH	Stool	*qnrS*, *qepA*, and *aac(6*′*)-Ib*	3	I	566	*dfrB4*	1	MW770326

129	UNC	Urine	*qnrS*	1	I	565	*dfrB4*	1	MW770327

UNC: Urology and Nephrology Center, GEH: Gastroenterology Hospital, MDICU: Microbiology Diagnostic Infection Control Unit, and -^1^: not sequenced.

## Data Availability

The data used to support the findings of this study are available from the corresponding author upon request.

## References

[1] Tenaillon O., Skurnik D., Picard B., Denamur E. (2010). The population genetics of commensal *Escherichia coli*. *Nature Reviews Microbiology*.

[2] Alharbi N. S., Khaled J. M., Kadaikunnan S. (2019). Prevalence of *Escherichia coli* strains resistance to antibiotics in wound infections and raw milk. *Saudi Journal of Biological Sciences*.

[3] Hooper D. C., Jacoby G. A. (2015). Mechanisms of drug resistance: quinolone resistance. *Annals of the New York Academy of Sciences*.

[4] Rodríguez-Martínez J. M., Machuca J., Cano M. E., Calvo J., Martínez-Martínez L., Pascual A. (2016). Plasmid-mediated quinolone resistance: two decades on. *Drug Resistance Updates*.

[5] Cocchi S., Grasselli E., Gutacker M., Benagli C., Convert M., Piffaretti J.-C. (2007). Distribution and characterization of integrons in *Escherichia coli* strains of animal and human origin. *FEMS Immunology & Medical Microbiology*.

[6] Cury J., Jové T., Touchon M., Néron B., Rocha E. P. (2016). Identification and analysis of integrons and cassette arrays in bacterial genomes. *Nucleic Acids Research*.

[7] Yang X., Zou W., Zeng J. (2017). Prevalence of antimicrobial resistance and integron gene cassettes in *Escherichia coli* isolated from yaks (*Poephagus grunniens*) in Aba Tibetan Autonomous Prefecture, China. *Microbial Pathogenesis*.

[8] Huang J., Lan F., Lu Y., Li B. (2020). Characterization of integrons and antimicrobial resistance in *Escherichia coli* sequence type 131 isolates. *The Canadian Journal of Infectious Diseases & Medical Microbiology*.

[9] Kang H. Y., Jeong Y. S., Oh J. Y. (2005). Characterization of antimicrobial resistance and class 1 integrons found in *Escherichia coli* isolates from humans and animals in Korea. *Journal of Antimicrobial Chemotherapy*.

[10] Odetoyin B. W., Labar A. S., Lamikanra A., Aboderin A. O., Okeke I. N. (2017). Classes 1 and 2 integrons in faecal *Escherichia coli* strains isolated from mother-child pairs in Nigeria. *PLoS One*.

[11] Tomova A., Ivanova L., Buschmann A. H., Godfrey H. P., Cabello F. C. (2018). Plasmid-mediated quinolone resistance (PMQR) genes and class 1 integrons in quinolone-resistant marine bacteria and clinical isolates of *Escherichia coli* from an aquacultural area. *Microbial Ecology*.

[12] Amin M., Dibachi S., Shahin M. (2017). Prevalence of class 1 integrons and plasmid-mediated qnr-genes among Enterobacter isolates obtained from hospitalized patients in Ahvaz, Iran. *Infezioni in Medicina, Le*.

[13] Seo K. W., Lee Y. J. (2019). Prevalence and characterization of plasmid mediated quinolone resistance genes and class 1 integrons among multidrug-resistant *Escherichia coli* isolates from chicken meat. *The Journal of Applied Poultry Research*.

[14] Bergey D. H., Krieg N. R., Holt J. G. (1984). *Bergey’s Manual of Systemic Bacteriology*.

[15] Clinical and Laboratory Standard Institute (2014). *CLSI; M100-S24 Performance Standards For Antimicrobial Susceptibility Testing. Twenty-four Informational Supplements*.

[16] Englen M. D., Kelley L. C. (2000). A rapid DNA isolation procedure for the identification of *Campylobacter jejuni* by the polymerase chain reaction. *Letters in Applied Microbiology*.

[17] FarajzadehSheikh A., Veisi H., Shahin M., Getso M., Farahani A. (2019). Frequency of quinolone resistance genes among extended-spectrum *β*-lactamase (ESBL)-producing *Escherichia coli* strains isolated from urinary tract infections. *Tropical Medicine and Health*.

[18] SAHIN S. (2020). Determination of the ciprofloxacin-resistant *Escherichia coli* isolated from chicken meat in Turkey. *Journal of the Hellenic Veterinary Medical Society*.

[19] Yoon M. Y., Kim Y. B., Ha J. S. (2020). Molecular characteristics of fluoroquinolone-resistant avian pathogenic *Escherichia coli* isolated from broiler chickens. *Poultry Science*.

[20] Goudarzi M., Azad M., Seyedjavadi S. S. (2015). Prevalence of plasmid-mediated quinolone resistance determinants and OqxAB efflux pumps among extended-spectrum *β*-lactamase producing *Klebsiella pneumoniae* isolated from patients with nosocomial urinary tract infection in Tehran, Iran. *Scientific*.

[21] Rizk D. E., El-Mahdy A. M. (2017). Emergence of class 1 to 3 integrons among members of Enterobacteriaceae in Egypt. *Microbial Pathogenesis*.

[22] Osińska A., Harnisz M., Korzeniewska E. (2016). Prevalence of plasmid-mediated multidrug resistance determinants in fluoroquinolone-resistant bacteria isolated from sewage and surface water. *Environmental Science and Pollution Research*.

[23] Taha S. A., Omar H. H., Hassan wH. (2019). Characterization of plasmid-mediated qnrA and qnrB genes among Enterobacteriaceae strains: quinolone resistance and ESBL production in Ismailia, Egypt. *Egyptian Journal of Medical Human Genetics*.

[24] Sanchez G. V., Adams S. J. E., Baird A. M. G., Master R. N., Clark R. B., Bordon J. M. (2013). *Escherichia coli* antimicrobial resistance increased faster among geriatric outpatients compared with adult outpatients in the USA, 2000-10. *Journal of Antimicrobial Chemotherapy*.

[25] Yang B., Yang F., Wang S. (2018). Analysis of the spectrum and antibiotic resistance of uropathogens in outpatients at a tertiary hospital. *Journal of Chemotherapy*.

[26] Seitz M., Stief C., Waidelich R. (2017). Local epidemiology and resistance profiles in acute uncomplicated cystitis (AUC) in women: a prospective cohort study in an urban urological ambulatory setting. *BMC Infectious Diseases*.

[27] Hayami H., Takahashi S., Ishikawa K. (2013). Nationwide surveillance of bacterial pathogens from patients with acute uncomplicated cystitis conducted by the Japanese surveillance committee during 2009 and 2010: antimicrobial susceptibility of *Escherichia coli* and *Staphylococcus saprophyticus*. *Journal of Infection and Chemotherapy: Official Journal of the Japan Society of Chemotherapy*.

[28] Australian Commission on Safety and Quality in Health Care (2014). *Preliminary Report on Antimicrobial Use and Resistance in Australia (AURA)*.

[29] Lee D. S., Lee S.-J., Choe H.-S. (2018). Community-acquired urinary tract infection by *Escherichia coli* in the era of antibiotic resistance. *BioMed Research International*.

[30] Strahilevitz J., Jacoby G. A., Hooper D. C., Robicsek A. (2009). Plasmid-mediated quinolone resistance: a multifaceted threat. *Clinical Microbiology Reviews*.

[31] Gururaju T., Kasturi T., Mallikarjuna reddy C. (2015). A study of antibiotic sensitivity pattern and detection of fluoroquinolones resistance to *Escherichia coli* from urinary tract infections. *International Journal of Current Microbiology and Applied Sciences*.

[32] Karimi Dehkordi M., Halaji M., Nouri S. (2020). Prevalence of class 1 integron in *Escherichia coli* isolated from animal sources in Iran: a systematic review and meta-analysis. *Tropical Medicine and Health*.

[33] Araújo B. F., Campos P. A. D., Royer S. (2017). High frequency of the combined presence of QRDR mutations and PMQR determinants in multidrug-resistant *Klebsiella pneumoniae* and *Escherichia coli* isolates from nosocomial and community-acquired infections. *Journal of Medical Microbiology*.

[34] Malek M. M., Amer F. A., Allam A. A., El-Sokkary R. H., Gheith T., Arafa M. A. (2015). Occurrence of classes I and II integrons in Enterobacteriaceae collected from Zagazig University Hospitals, Egypt. *Frontiers in Microbiology*.

[35] Singh N. S., Singhal N., Kumar M., Virdi J. S. (2021). High prevalence of drug resistance and class 1 integrons in *Escherichia coli* isolated from river Yamuna, India: a serious public health risk. *Frontiers in Microbiology*.

[36] Deng Y., Bao X., Ji L. (2015). Resistance integrons: class 1, 2 and 3 integrons. *Annals of Clinical Microbiology and Antimicrobials*.

[37] Zeeshan Khan F., Nawaz T., Mirani Z. A., Khan S., Raza Y., Kazmi S. U. (2018). Study of class 1 integrons in multidrug-resistant uropathogenic *Escherichia coli* isolated from different hospitals in Karachi. *Iranian Journal of Basic Medical Sciences*.

[38] Jones-Dias D., Manageiro V., Ferreira E. (2016). Architecture of class 1, 2, and 3 integrons from gram negative bacteria recovered among fruits and vegetables. *Frontiers in Microbiology*.

[39] Esmaeel N. E., Gerges M. A., Hosny T. A., Ali A. R., Gebriel M. G. (2020). Detection of chromosomal and plasmid-mediated quinolone resistance among *Escherichia coli* isolated from urinary tract infection cases; Zagazig university hospitals, Egypt. *Infection and Drug Resistance*.

[40] Khalil M., Elsherif R., Ghaith D. (2017). Quinolone resistance detection by PCR-RFLP and multiplex-PCR among extended- spectrum *β*- lactamase producing *Enterobacteriaceae*. *International Journal of Clinical & Medical Microbiology*.

[41] Al-Hasnawy H. H., Jodi M. R., Hamza H. J. (2018). Molecular characterization and sequence analysis of plasmid-mediated quinolone resistance genes in extended-spectrum beta-lactamases producing uropathogenic *Escherichia coli* in Babylon Province, Iraq. *Reviews in Medical Microbiology*.

[42] Malekzadegan Y., Rastegar E., Moradi M., Heidari H., Sedigh Ebrahim-Saraie H. (2019). Prevalence of quinolone-resistant uropathogenic *Escherichia coli* in a tertiary care hospital in south Iran. *Infection and Drug Resistance*.

[43] Al-Agamy M. H., Aljallal A., Radwan H. H., Shibl A. M. (2018). Characterization of carbapenemases, ESBLs, and plasmid-mediated quinolone determinants in carbapenem-insensitive *Escherichia coli* and *Klebsiella pneumoniae* in Riyadh hospitals. *Journal of Infection and Public Health*.

[44] Kao C.-Y., Wu H.-M., Lin W.-H. (2016). Plasmid-mediated quinolone resistance determinants in quinolone-resistant *Escherichia coli* isolated from patients with bacteremia in a university hospital in Taiwan, 2001-2015. *Scientific Reports*.

[45] Kotb D. N., Mahdy W. K., Mahmoud M. S., Khairy R. M. M. (2019). Impact of co-existence of PMQR genes and QRDR mutations on fluoroquinolones resistance in Enterobacteriaceae strains isolated from community and hospital acquired UTIs. *BMC Infectious Diseases*.

[46] Yang J., Luo Y., Cui S., Wang W., Han L. (2011). Diverse phenotypic and genotypic characterization among Clinical Klebsiella pneumoniae and Escherichia coli isolates carrying plasmid-mediated quinolone resistance determinants. *Microbial Drug Resistance*.

[47] Hamed S. M., Aboshanab K. M. A., El-Mahallawy H. A., Helmy M. M., Ashour M. S., Elkhatib W. F. (2018). Plasmid-mediated quinolone resistance in gram-negative pathogens isolated from cancer patients in Egypt. *Microbial Drug Resistance*.

[48] Khalifa H. O., Soliman A. M., Ahmed A. M. (2019). High prevalence of antimicrobial resistance in gram-negative bacteria isolated from clinical settings in Egypt: recalling for judicious use of conventional antimicrobials in developing nations. *Microbial Drug Resistance*.

[49] Li B., Chen Y., Wu Z., Zhao Z., Wu J., Cao Y. (2018). Prevalence of plasmid-mediated quinolone resistance genes among *Escherichia coli* in the gut of healthy people in Fuzhou, China. *Annals of Laboratory Medicine*.

[50] El-Badawy M. F., Tawakol W. M., El-Far S. W. (2017). Molecular identification of aminoglycoside-modifying enzymes and plasmid-mediated quinolone resistance genes among *Klebsiella pneumoniae* clinical isolates recovered from Egyptian patients. *International Journal of Microbiology*.

[51] Szabó O., Gulyás D., Szabó N., Kristóf K., Kocsis B., Szabó D. (2018). Plasmid-mediated quinolone resistance determinants in Enterobacteriaceae from urine clinical samples. *Acta Microbiologica et Immunologica Hungarica*.

[52] Zaki M. E. S., El Salam M. A., Faried O. A. (2021). Study of plasmid-mediated quinolone resistance in *Escherichia coli* from nosocomial urinary tract infections. *Infectious Disorders-Drug Targets*.

[53] Azargun R., Sadeghi M. R., Soroush Barhaghi M. H. (2018). The prevalence of plasmid-mediated quinolone resistance and ESBL-production in Enterobacteriaceae isolated from urinary tract infections. *Infection and Drug Resistance*.

[54] Majlesi A., Kakhki R. K., Mozaffari Nejad A. S. (2018). Detection of plasmid-mediated quinolone resistance in clinical isolates of Enterobacteriaceae strains in Hamadan, West of Iran. *Saudi Journal of Biological Sciences*.

[55] Akya A., Chegene Lorestani R., Elahi A., Ghadiri K. (2017). The impact of mutations in topoisomerase genes and the plasmid-mediated quinolone resistance (PMQR) determinants on the resistance to fluoroquinolones in *Klebsiella pneumoniae*. *Archives of Clinical Infectious Diseases*.

[56] Robicsek A., Strahilevitz J., Jacoby G. A. (2006). Fluoroquinolone-modifying enzyme: a new adaptation of a common aminoglycoside acetyltransferase. *Nature Medicine*.

[57] Yang H. Y., Nam Y. S., Lee H. J. (2014). Prevalence of plasmid-mediated quinolone resistance genes among ciprofloxacin-nonsusceptible *Escherichia coli* and *Klebsiella pneumoniae* isolated from blood cultures in Korea. *The Canadian Journal of Infectious Diseases & Medical Microbiology*.

[58] Malekzadegan Y., Rastegar E., Moradi M., Heidari H., Sedigh Ebrahim-Saraie H. (2019). Prevalence of quinolone-resistant uropathogenic *Escherichia coli* in a tertiary care hospital in south Iran [Response to letter]. *Infection and Drug Resistance*.

[59] Tao Y., Zhou K., Xie L. (2020). Emerging coexistence of three PMQR genes on a multiple resistance plasmid with a new surrounding genetic structure of qnrS2 in *E. coli* in China. *Antimicrobial Resistance & Infection Control*.

[60] Jacoby G. A., Strahilevitz J., Hooper D. C. (2014). Plasmid-mediated quinolone resistance. *Microbiology Spectrum*.

[61] Zhang S., Yang H., Rehman M. U. (2019). Class 1 integrons as predominant carriers in *Escherichia coli* isolates from waterfowls in Hainan, China. *Ecotoxicology and Environmental Safety*.

[62] Moura A., Henriques I., Ribeiro R., Correia A. (2007). Prevalence and characterization of integrons from bacteria isolated from a slaughterhouse wastewater treatment plant. *Journal of Antimicrobial Chemotherapy*.

[63] Dawes F. E., Kuzevski A., Bettelheim K. A., Hornitzky M. A., Djordjevic S. P., Walker M. J. (2010). Distribution of class 1 integrons with IS26-mediated deletions in their 3′-conserved segments in *Escherichia coli* of human and animal origin. *PLoS One*.

[64] Japoni-Nejad A., Farshad S., van Belkum A., Ghaznavi-Rad E. (2013). Novel cassette array in a class 1 integron in clinical isolates of *Acinetobacter baumannii* from central Iran. *International Journal of Medical Microbiology*.

[65] Kargar M., Mohammadalipour Z., Doosti A., Lorzadeh S., Japoni-Nejad A. (2014). High prevalence of class 1 to 3 integrons among multidrug-resistant diarrheagenic *Escherichia coli* in southwest of Iran. *Osong Public Health and Research Perspectives*.

[66] Chen J., Su Z., Liu Y. (2009). Identification and characterization of class 1 integrons among *Pseudomonas aeruginosa* isolates from patients in Zhenjiang, China. *International Journal of Infectious Diseases*.

[67] Kaushik M., Kumar S., Kapoor R. K., Virdi J. S., Gulati P. (2018). Integrons in Enterobacteriaceae: diversity, distribution and epidemiology. *International Journal of Antimicrobial Agents*.

[68] Su H.-C., Ying G.-G., Tao R., Zhang R.-Q., Zhao J.-L., Liu Y.-S. (2012). Class 1 and 2 integrons, sul resistance genes and antibiotic resistance in *Escherichia coli* isolated from Dongjiang River, South China. *Environmental Pollution*.

[69] Kubomura A., Sekizuka T., Onozuka D. (2020). Truncated class 1 integron gene cassette arrays contribute to antimicrobial resistance of diarrheagenic *Escherichia coli*. *BioMed Research International*.

[70] Mazel D. (2006). Integrons: agents of bacterial evolution. *Nature Reviews Microbiology*.

[71] Leverstein–van Hall M. A., Box A. T. A., Blok H. E. M., Paauw A., Fluit A. C., Verhoef J. (2002). Evidence of extensive interspecies transfer of integron‐mediated antimicrobial resistance genes among multidrug‐resistant Enterobacteriaceae in a clinical setting. *The Journal of Infectious Diseases*.

[72] Zhang H., Shi L., Li L. (2004). Identification and characterization of class 1 integron resistance gene cassettes among Salmonella strains isolated from healthy humans in China. *Microbiology and Immunology*.

[73] Chang L.-L., Chang T.-M., Chang C.-Y. (2007). Variable gene cassette patterns of class 1 integron-associated drug-resistant *Escherichia coli* in Taiwan. *The Kaohsiung Journal of Medical Sciences*.

[74] White P. A., McIver C. J., Deng Y.-M., Rawlinson W. D. (2000). Characterisation of two new gene cassettes, aadA5 and dfrA17. *FEMS Microbiology Letters*.

[75] Lindstedt B.-A., Heir E., Nygard I., Kapperud G. (2003). Characterization of class I integrons in clinical strains of *Salmonella enterica* subsp. *enterica* serovars *Typhimurium* and *Enteritidis* from Norwegian hospitals. *Journal of Medical Microbiology*.

[76] Guerra B., Junker E., Schroeter A., Malorny B., Lehmann S., Helmuth R. (2003). Phenotypic and genotypic characterization of antimicrobial resistance in German *Escherichia coli* isolates from cattle, swine and poultry. *Journal of Antimicrobial Chemotherapy*.

[77] Canal N., Meneghetti K. L., de Almeida C. P., da Rosa Bastos M., Otton L. M., Corção G. (2016). Characterization of the variable region in the class 1 integron of antimicrobial-resistant *Escherichia coli* isolated from surface water. *Brazilian Journal of Microbiology*.

